# Difficulties in the Management of the Social Evil

**Published:** 1902-02

**Authors:** George R. White

**Affiliations:** Savannah, Ga.


ATLANTA

### Journal-Record of Medicine.

**Successor to Atlanta Medical and Surgical Journal, Established 1855, and Southern Medical Record, Established 1870.**

VOL. III. FEBRUARY, 1902. No. 11.

BERNARD WOLFF, M.D., EDITOR, Nos. 319–20 Prudential.

Published Monthly.

M. B. HUTCHINS, M.D., BUSINESS MANAGER, No. 64 Marietta St.

### *ORIGINAL COMMUNICATIONS.*

### DIFFICULTIES IN THE MANAGEMENT OF THE SOCIAL EVIL.*

BY GEORGE R. WHITE, M.D.,
SAVANNAH, GA.

In a society of this kind we need not apologize for bringing up a subject as old as the race, the social evil, and the diseases resulting therefrom. It is a matter that has attracted the attention of thinking men of all ages, and remedies have been advanced varying in kind, from our present method of ignoring the evil entirely to the law of the ancient Hebrews which required both parties to be stoned to death. There seems to have been no great success in managing the evil under any system, and it is now much the same as it always has been. The century upon which we have just entered is one in which the impossible of other centuries will be accomplished, and perhaps some wise man will point out the way in which this greatest of evils may be relieved or eradicated.

* Read at the Georgia State Sociological Society Meeting. Atlanta, Ga., Dec. 13-14, 1901.

The purpose of this paper is to speak of the subject from the medical side, especially the diseases associated with it and the difficulties of controlling them, without being able to suggest any very radical remedies.

Leaving out the less important ailments, there are two highly contagious venereal diseases which are spreading, among the innocent and guilty alike, and working incalculable harm in society without any efficient effort being made to control them. Each of the diseases is of long duration, often does not greatly incapacitate the sufferer, and presents peculiar features which make it difficult to manage. Syphilis, the most feared of these troubles, is a disease the nature of which is not yet known, but it is doubtless of bacterial origin. It is divided into three stages. The primary stage is characterized by an ulcer at the site of infection, and lasts two or three months; in the secondary stage there is an eruption over the entire body and superficial ulcers about the mouth and genitals; this is the most infectious period, and lasts from a few months to two years; then the disease may be cured or go on to a third stage, which lasts the remainder of the victim's life and produces a great variety of troubles, including deep ulcers, tumors of the different organs, especially the brain, apoplexy, necroses of bone, locomotor ataxia and other spinal cord disease. The disease is transmitted to the offspring by either father or mother. The children, however, seldom grow up, but after a miserable existence of months or years die of ulceration or marasmus. The first and second stages are the times of infection, not only by the genitals, but by the mouth as well. The diseased individuals go about the community often a year or more, scattering infection broadcast by contaminating spoons, forks, drinking-cup, coins, pipes, towels, bedding, or any other object with which they may come in contact, besides kissing their friends and their friends' children, and inflicting on innocent wife, or husband if they have one. Syphilis in the innocent is an every-day occurrence and forms a considerable proportion of those suffering from the disease. Those who realize what they have and are careful about not spreading it are the exception. The disease is most common in the ignorant and careless, who are utterly indifferent to the amount of misery they may cause by spreading it, and they often boast that they got the disease from somebody and are going to pay it back with interest.

Gonorrhea, while popularly supposed to be a mild disease, is more difficult to manage and produces even greater trouble than syphilis. It is a disease caused by bacteria that can be isolated, grown in a culture tube, and reinocculated, producing the disease. The bacteria are found in pairs; they are spherical in form and flattened on the side next their fellow. From this peculiarity they are called diplococci. They are easily killed by heat, antisceptics, or drying, but in their natural habitat in the mucous membrane of the genital tract they resist all known methods of getting rid of them. They live under the epithelium and down in the crypts and glands, where remedial agents cannot reach them. After an individual has been infected the diplococci do not disappear with the disappearance of the symptoms of the disease, but live on in a quiescent state, ready to take on new activity and infect one or both parties. While the disease is easy to recognize in its acute stage both by the symptoms and the microscope, after the acute stage has subsided it is practically impossible to determine whether the power of infection is still present. The diplococci become few in number, live deep in the tissues, and lose somewhat their diplococci form, and are easily mistaken for the common staphylococcus or some of the other diplococci which inhabit these parts.

The disease in the male is often of a mild type, lasting from four to eight weeks and remaining latent an indefinite period. It is, however, liable to produce several grave complications, the most common of which is an infection of the knee or some other large joint with complete and permanent disability, and a fatal result is not uncommon from involvement of the kidneys. In the female there is a very different course. The acute stage is frequently wanting, especially if the infection is from a latent case; but the disease extends upward into the uterus, tubes, ovaries, and sensitive structures in the pelvis, producing chronic inflammations, suppuration and permanent destruction of these sensitive structures, with excruciating pain, especially at the menstrual epoch, and leading to nervous break-down and sterility.

It is only too common an occurence to see a young woman starting out on her married life with every prospect of health and happiness, fall a victim to an uncured case of gonorrhea in her husband, and in a few months become a physical and nervous wreck, destined to suffer for years the torments of the *inferno*, and spend the remainder of her life as a childless invalid, hiding the real cause of her trouble from her friends, or else submit to the removal of her sexual organs.

This disease also attacks the eyes, especially in children at birth, producing severe inflammation and often blindness. An adult rarely recovers from an attack of gonorrheal ophthalmia without losing his eyesight. Statistics show that about half of the inmates of our blind asylums have lost their eyesight from this cause.

Our present attitude toward these diseases is to look upon them perhaps as a punishment for crime, and let them absolutely alone. The diseased are allowed to go about infecting guilty and innocent alike. Our hospitals are usually closed against those who would enter and be cured; and there is general ignorance as to the true nature and seriousness of the diseases. This is not a satisfactory method of dealing with a great evil, but no other method has produced much better results. Municipal inspection and control is practically the only reasonable method that has been extensively tried of late years. This requires that the evil be recognized, given a legal status and protection; and a hospital be maintained where the diseased shall be sent and kept, against their will, if necessary, until they are no longer infectious. The difficulties in the way of this system are many. In the first place, the nature of the diseases would require the infected individual to be shut up many months at a time and be returned at each new outbreak, so that many of them would spend the greater part of their time in the institution. Moreover, the difficulty or impossibility of saying just when a patient is no longer infectious would either keep the inmates shut up unnecessarily long, or send them out as a source of infection, to the discredit of the system and to the chagrin of the patron, who would expect protection while the business is under the control of the city. It would be manifestly unreasonable to shut up only the licensed women and leave their diseased partners free to infect the new recruits as they come on till the institutions were full or the supply exhausted, and at the same time break up one of the great safeguards against the evil, namely, the tendency of the vicious to flock together and the diseased consort only with the diseased.

Nobody thinks seriously of capturing all the infected men and shutting them up in an institution for several months ; our accommodations would be hardly large enough.

The chief objection to municipal regulation is that this vilest of trades is carried on under the protection of the law and an implied guarantee of freedom from infection which at once tends to break down the moral and social restraint holding it in check, and makes the evil more attractive to both sexes.

Only a limited number, however, would take the protection that the city offers, and there would be two systems: one in which the evil exists in its present illegal form undiminished in extent and viciousness, and the other, that would serve but little purpose except as a recruiting place for the old system.

Legal control in Vienna and Paris are anything but successes, for the reasons mentioned above, and the evil there is as great as anywhere in the world.

There are, however, several things that can be done.

Some provision should be made for receiving and keeping all diseased who ask for hospital treatment.

The Health Board should assume power to shut up the specially vicious and dangerous cases till they recover.

There should be correct teaching among the laity upon several points: first, that sexual indulgence is not necessary to health, though there is a popular belief to the contrary; second, that syphilis is an exceedingly contagious disease and all care should be taken to prevent its spread, and, finally, that gonorrhea is not cured when the symptoms subside, but remains latent and may start up a fresh disease months after a supposed cure.

Should a law against the marrying of the insane and unfit be enacted as recommended by this society, it ought also to contain a clause forbidding the marriage of syphilitics and those recently affected by gonorrhea.

